# L1pred: A Sequence-Based Prediction Tool for Catalytic Residues in Enzymes with the L1-logreg Classifier

**DOI:** 10.1371/journal.pone.0035666

**Published:** 2012-04-27

**Authors:** Yongchao Dou, Jun Wang, Jialiang Yang, Chi Zhang

**Affiliations:** 1 School of Biological Sciences, Center for Plant Science and Innovation, University of Nebraska, Lincoln, Nebraska, United States of America; 2 Scientific Computing Key Laboratory of Shanghai Universities, Shanghai, People’s Republic of China; 3 Department of Mathematics, Shanghai Normal University, Shanghai, People’s Republic of China; 4 MPI-Institute of Computational Biology, Chinese Academy of Sciences, Shanghai, People’s Republic of China; Miami University, United States of America

## Abstract

To understand enzyme functions, identifying the catalytic residues is a usual first step. Moreover, knowledge about catalytic residues is also useful for protein engineering and drug-design. However, to experimentally identify catalytic residues remains challenging for reasons of time and cost. Therefore, computational methods have been explored to predict catalytic residues. Here, we developed a new algorithm, L1pred, for catalytic residue prediction, by using the L1-logreg classifier to integrate eight sequence-based scoring functions. We tested L1pred and compared it against several existing sequence-based methods on carefully designed datasets Data604 and Data63. With ten-fold cross-validation, L1pred showed the area under precision-recall curve (AUPR) and the area under ROC curve (AUC) of 0.2198 and 0.9494 on the training dataset, Data604, respectively. In addition, on the independent test dataset, Data63, it showed the AUPR and AUC values of 0.2636 and 0.9375, respectively. Compared with other sequence-based methods, L1pred showed the best performance on both datasets. We also analyzed the importance of each attribute in the algorithm, and found that all the scores contributed more or less equally to the L1pred performance.

## Introduction

Enzymes are very important because they act as catalysts for almost all chemical reactions in a cell to make the reaction rates sufficient for life. Identifying catalytic residues of enzymes is a crucial step towards understanding their functions. The knowledge on catalytic residues can further help design novel proteins with new functions and hence be useful for drug-design. Despite the importance, the number of proteins with known catalytic sites compared with the huge number of enzymes is still small, as it is often expensive and time consuming to experimentally identify catalytic residues. Fortunately, computational methods have become an important tool to predict catalytic residues with more and more annotated enzymes available.

In the past decade and a half, many computational methods have been developed to predict catalytic residues on given enzymes. The forerunners only considered protein sequence conservation information [1]–[12]. Prediction methods were then improved by incorporating phylogenetic motifs [13], [14], phylogenetic trees [15], [16], predicted structural information [17], and amino acids stereo-chemical properties [18]–[20] with conservation information. With increasing number of solved protein structures, structural information was also taken into account by many algorithms, however, which were limited only to proteins with known structures [21]–[31]. Meanwhile, Brylinski *et al.* developed a method to recognize protein active sites based on the analysis of hydrophobicity distribution in protein molecules [32]. In recent years, machine learning algorithms, such as Support Vector Machine-based (SVM) and Neural Network-based (NN), were used to develop new catalytic residue prediction methods [33]–[40]. The machine-learning algorithms can easily integrate various chemical and physical features of residues, such as sequence conservation, residue types, cumulative hydrophobicity, secondary structure, and relative solvent accessibility. For instance, Gutteridge *et al.* [33] used NN to incorporate six attributes extracted from both protein sequences and structures. Petrova and Wu [35] developed a similar method but using SVM. Zhang *et al.* [37] proposed an SVM-based method, called CRpred, which used sequence-derived attributes only. Youn reviewed several frequently used features and ranked their performance based on their ability to distinguish catalytic residues from non-catalytic ones; the top-ranked features are sequence conservation, structural conservation, uniqueness of a residue’s structural environment, solvent accessibility, and residue hydrophobicity [36]. The flourishing efforts demonstrated promising potentials of computational methods on this research front, yet higher prediction accuracy is still needed for better performance.

In this manuscript, we developed a tool to predict enzyme catalytic residues. This tool is called L1pred because it uses the L1-logred classifier, which is an implementation of the interior-point method for L1-regularized logistic regression [41]. Eight scoring functions used by L1pred to abstract protein sequence chemical/physical characteristics are residue type (RT), overlapping properties (OP), averaged cumulative hydrophobicity (ACH), predicted protein secondary structure (SS), predicted accessible surface area (ASA), Jensen-Shannon divergence (JSD) conservation score, the combination of relative entropy of Venn diagram and JSD conservation score (VJSD), and Consurf score. We compared our method with others, such as JSD [5], VJSD [19], Consurf [42] and CRpred [37], and L1pred was shown to have the highest AUPR and AUC value for the same datasets. The curated datasets, the trained model, and the source code files are available at http://sysbio.unl.edu/L1pred.

## Results

### Results on the Dataset Data604

**Figure 1:**
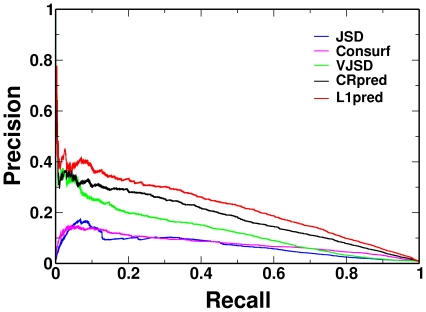
PR curves of five methods on the Data604 dataset.

The parameters of L1pred were trained on the dataset Data604. The performance of L1pred achieved the optimal point at window-size = 6 and 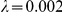; the corresponding maximal AUPR and AUC are 0.2198 and 0.9494, respectively. In the rest of the study, we applied window-size = 6 and 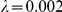 as the default setting. Our method was compared against four sequence-based methods JSD, VJSD, Consurf, and CRpred, on the dataset Data604. JSD is a sequence conservation based method which uses amino acid position specific frequencies [5]. VJSD takes both stereo-chemical property and residues frequencies into account [19]. Consurf incorporates both sequence conservation information and evolutionary relations among the protein and its homologous sequences [42]. CRpred is an SVM based method which takes five types of attributes into account, including (1) residue type, (2) position specific scoring matrix (PSSM), (3) Shannon entropy computed over the weighted observed percentages (WOP) vector, (4) averaged cumulative hydrophobicity and (5) catalytic resides pairs [37]. Of the four methods used for comparison, JSD, VJSD, and Consurf do not need a training procedure, while CRpred does and therefore it was trained using the same procedure as our method. The optimal parameters of CRpred were obtained from [37], and we tested CRpred with the same ten-fold cross validation procedure as L1pred. The comparison results are shown in Table 1. L1pred shows the best values in terms of both AUPR and AUC, in detail, resulting to AUPR = 0.2198 and AUC = 0.9494. Moreover, L1pred is significantly better than the other four methods (with P-value = 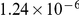
<0.05), according to the ROC significance test. Figure 1 shows the PR curves for all five methods, and the PR curve of L1pred is constantly higher than that of the other PR curves in the whole range of recall rate.

**Table 1:** Performance on the dataset Data604.

Method	AUPR	AUC	Recall	Precision
JSD	0.0692	0.8443	0.3299	0.1016
Consurf	0.0778	0.8969	0.3515	0.0944
VJSD	0.1300	0.8700	0.3724	0.1593
CRpred	0.1819	0.9338	0.3805	0.2310
L1pred	0.2198	0.9494	0.3741	0.2752

### Results on the Independent Test Dataset Data63

**Figure 2:**
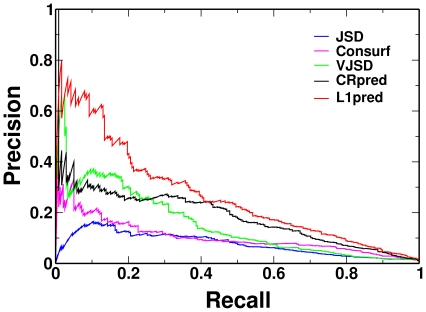
PR curves of five methods on the Data63 dataset.

All chosen methods were also compared using the independent test set, Data63, and the results were in broad agreement with what found on the dataset Data604. For L1pred and CRpred, their trained models were generated on the whole Data604 dataset. All results are shown in Table 2, and L1pred shows the best performance. For example, L1pred has the highest values of AUPR and AUC of 0.2636 and 0.9375, respectively. We also tested the statistical significance among different methods in terms of the AUC values. L1pred is significantly better than the other methods; all comparisons showed P-values 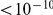, except with CRpred method (P-value = 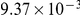), but it is still significant for the cutoff of P-value = 0.05. From the PR curve, shown in Figure 2, one may find that the PR curve of L1pred is notably higher than that of CRpred, the second best method. Especially, if using recall rate = 0.1, the precision of L1pred is more than 60%, while the second best performer is less than 40%. However, all precisions drop fast; at the maximal F-measure point, *i.e.* recall =  0.3571, even the precision of L1pred drops to only 0.3257. These results indicate that L1pred achieves comparable performance on independent dataset with the trained parameters.

**Table 2:** Performance on the dataset Data63.

Method	AUPR	AUC	Recall	Precision
JSD	0.0759	0.8410	0.4160	0.1061
Consurf	0.1019	0.8876	0.2017	0.1644
VJSD	0.1520	0.8599	0.3109	0.2349
CRpred	0.1809	0.9201	0.4244	0.2446
L1pred	0.2636	0.9375	0.3571	0.3257

CRpred and L1pred have different attribute sets and classifiers. Additional analysis was conducted to figure out which one is essential in prediction. We applied L1-logred classifier to the attribute sets of CRpred method (CRpred-L1) and SVM to attributes of L1pred (L1pred-SVM). All parameters were optimized as the same procedure described in the section of Methods. For the dataset Data604 with ten-fold cross validation, the AUC value of CRpred-L1 is 0.9341, which is approximately equal to that of CRpred, 0.9338. L1pred and L1pred-SVM also have close AUC values on the dataset Data604; they are 0.9494 and 0.9480, respectively. The situation for the dataset Data63 is similar as well. These results indicate that the combination of those eight attributes used by L1pred plays important role in the improvement of prediction performance.

Moreover, L1pred is more efficient than other machine learning methods, *e.g.* the SVM-based CRpred method, because L1-logred is a fast classifier. Table 3 shows the result of the comparison between L1pred and CRpred in terms of computing time for training and testing. L1pred is about 40 times faster than CRpred in both training and predicting.

**Table 3:** Computing time of L1pred and CRpred methods.

Method	AUPR	AUC	Recall	Precision
JSD	0.0759	0.8410	0.4160	0.1061
Consurf	0.1019	0.8876	0.2017	0.1644
VJSD	0.1520	0.8599	0.3109	0.2349
CRpred	0.1809	0.9201	0.4244	0.2446
L1pred	0.2636	0.9375	0.3571	0.3257

### Results on the Dataset EF-family

L1pred and CRpred are applied on the dataset EF-family with the same ten-fold cross validation procedure. For CRpred, the encoded feature vector of each protein was directly downloaded from their web site [37]. This dataset has been used by Youn *et al.* to test their structure-based method [36], which integrated several different types of attributes, including structural conservation, B-factor, solvent accessibility, and sequence conservation *etc.*

From Table 4, which shows all results on the dataset EF-family, one may find that the overall results are similar to that on both Data604 and Data63. Specifically, the values of AUPR and AUC of L1pred, 0.2589 and 0.9372, are higher than those of CRpred. The difference between L1pred and CRpred is significant for ROC, with a P-value of 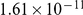. Moreover, L1pred is also slightly better than Youn’s method in terms of AUC. The result of Youn’s method on the dataset of EF-family was obtained directly from their publication [36].

**Table 4:** Performance on the dataset EF-family.

Method	AUPR	AUC	Recall	Precision
JSD	0.0841	0.8543	0.0886	0.5522
Consurf	0.0969	0.8767	0.1229	0.3048
VJSD	0.1695	0.8873	0.2333	0.2756
CRpred	0.2256	0.9118	0.2853	0.3838
Youn	N/A	0.9298	0.5702	0.1851
L1pred	0.2589	0.9372	0.4478	0.2862

### Importance of Different Features

To understand which attributes of all eight different scores play more important roles, we removed them one by one and repeated the same training and validation procedure on the dataset Data604. The results are shown in Table 5. One may find that the omission of any score leads to some changes in performance, but none was significant. The largest drop occurred when the Consurf score was turned off. We therefore concluded that all eight attributes are almost equally important for L1pred, but the Consurf score is slightly more important than all others.

**Table 5:** Performance of L1pred by removing attributes one by one.

Method	AUPR	AUC	Recall	Precision
no-Consurf	0.1688	0.9282	0.3854	0.2125
no-SS	0.2119	0.9467	0.4559	0.2440
no-RT	0.2128	0.9492	0.4370	0.2455
no-ACH	0.2129	0.9486	0.4736	0.2313
no-VJSD	0.2140	0.9488	0.4392	0.2466
no-JSD	0.2167	0.9492	0.4623	0.2422
no-ASA	0.2175	0.9494	0.3947	0.2640
no-OP	0.2184	0.9487	0.4128	0.2607
L1pred	0.2198	0.9494	0.3741	0.2752

**Figure 3:**
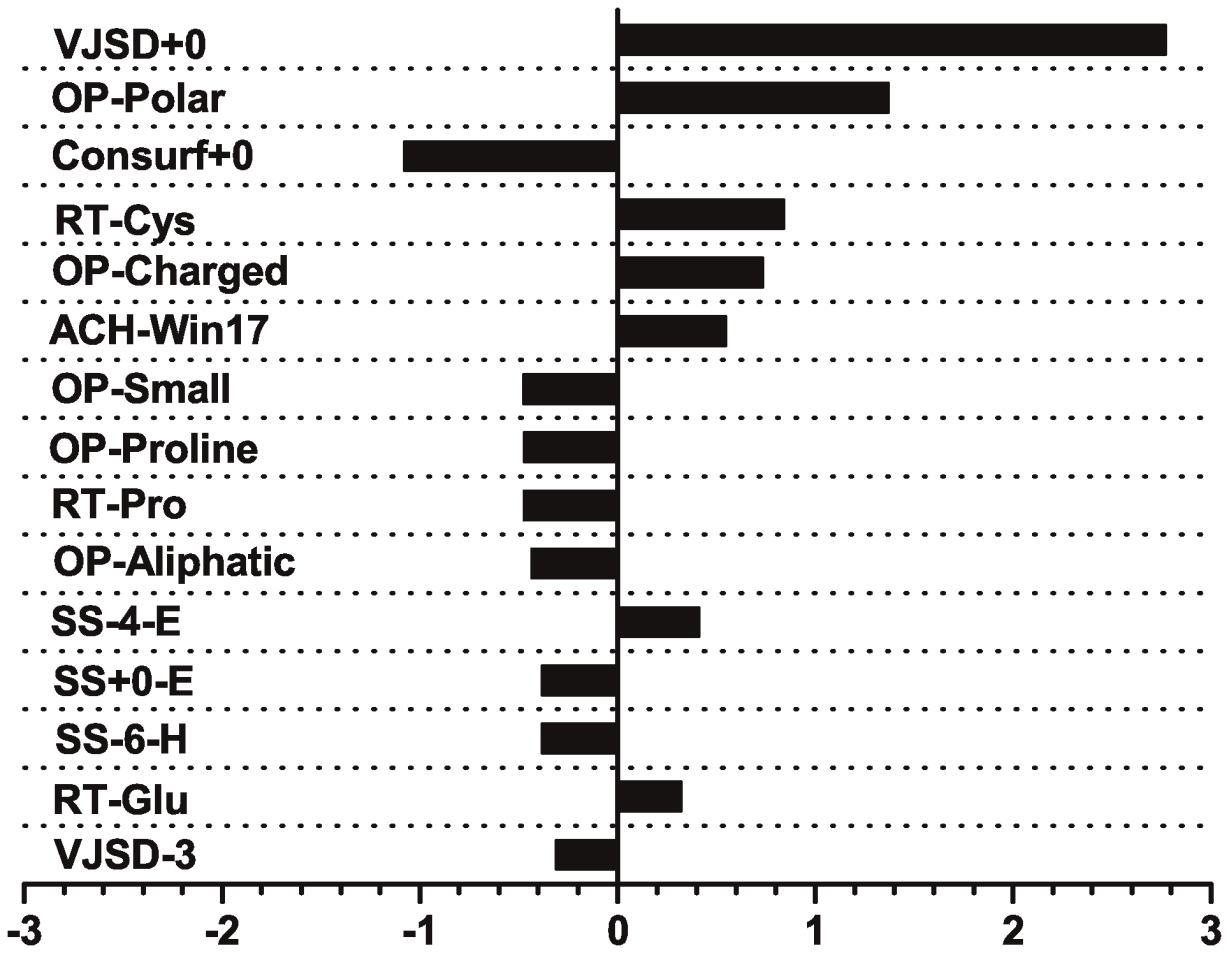
Weights of the top fifteen features on the Data604 dataset.

We also extracted the weight vector of the trained model on the whole Data604 dataset. The top 15 weighted bits are shown in Figure 3 in which, for example, SS-4-E denotes the SS attribute of the beta strand at the 4th position on the N-terminal side of the central bit in a sliding window. The similar notations are applied for the other features, and 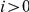 represents positions towards C- terminal, 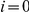 represents the central residue and 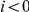 represents positions towards N-terminal. We found that VJSD+0 has the largest weight, which means the stereo-chemical characteristics are correctly reflected by this scoring function, and the majority of catalytic residues can be distinguished by this feature. In addition, being a Cys residue (RT-Cys) and/or a charged/polar residue (OP-Polar, OP-Charged) are important features for catalytic sites, which agrees with the statistical results [43]. In the trained model, the Consurf score of position 0 is also important for catalytic residues prediction as ranked on the third position. Assigning a large weight to ACH-Win17 indicates that the mean hydrophobicity of 16 residues around the catalytic residues plays an important role for catalytic functions. These results suggest that L1pred can extract the most useful chemical/physical characteristics of catalytic residues by the training procedure.

### Case Studies

**Figure 4:**
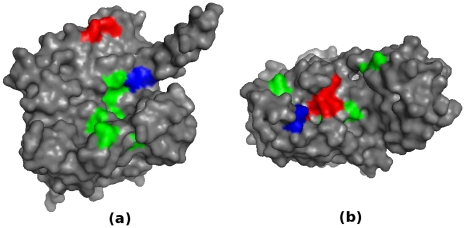
Prediction results of L1pred on a dehydrogenase (a) and an asparaginase (b) Red: true positive, blue: false negative, and green: false positive.

We randomly selected two enzymes from our datasets as examples to show the prediction performance of L1pred; they are a dehydrogenase (PDB ID: 1A05 chain A) and an asparaginase (PDB ID: 3ECA chain A). There are three catalytic residues for the dehydrogenase (140Y, 190L, and 222D) and five for the asparaginase (12T, 25Y, 89T, 90D, and 162L). Prediction results of L1pred are shown in Figure 4. For each enzyme, true catalytic residues and 10 top-ranked residues are shown in colors; correctly predicted catalytic residues are shown in red, missed catalytic residues (false negative) in blue, and the resides predicted by L1pred but not true catalytic residues (false positive) in green. Two out of three catalytic residues were correctly predicted for the dehydrogenase and four out of five for the asparaginase. Both cases indicate that L1pred can discover more than 60% catalytic residues with recall = 4%, as the lengths of those enzymes are both more than 300 amino acids.

## Discussion

We applied the L1-logreg classifier with eight attributes to predict enzyme catalytic residues. The attributes, VJSD, overlapping properties, and Consurf score, are newly introduced to the solution of catalytic residue prediction. With the ten-fold cross validation on the dataset Data604 and directly application on the independent test set Data63, L1pred showed the best performance among chosen algorithms. The AUC values of L1pred on the dataset Data604 and Data63 are 0.9494 and 0.9375, respectively, which are significantly higher than other prediction methods (P- value<0.05). The test on the EF-family dataset confirms that this method performs better than existing methods, including the structure-based one. In all eight attributes, Consurf, SS, RT, and averaged cumulative hydrophobicity play slightly more important roles than the other attributes. The scoring functions of Consurf and VJSD used in this manuscript can be combined with structural information to improve catalytic residue prediction. Further analysis indicates that the improvement made by L1pred is mainly due to the combination of informative attributes, instead of the classifier. L1-logreg classifier is not necessary to have better performance in catalytic residue prediction than SVM, but it is efficient and hence competent for genome-wide analyses, where speed is an issue. In the future, we will test additional scoring functions to further improve the prediction performance, and extend the platform developed for this project to other applications, such as protein phosphorylation site prediction.

## Materials and Methods

### Datasets

We collected our data from two sources: the datasets created by Zhang *et al.* [37] and the Catalytic Site Atlas (CSA) dataset [44]. By mixing the CSA and the eight datasets from Zhang *et al.* [37], (namely, EF-family, EF-fold, EF-superfamily, HA-superfamily, NN, PC, T-124, and T-37), we generated two new datasets. Since all enzymes in our datasets have structures in PDB, we first compared their sequences with the sequences of the structures in PDB [45]. If two sequences are not identical, this enzyme was discarded. For the remaining protein sequences, we clustered them using Blastclust [46] with sequence identity 30% and coverage 60%. A total of 667 clusters were returned, 604 of which have single members and 63 have multiple ones. Those 604 chains with sequence similarity lower than 30.0% to the other chains were selected as a dataset and named Data604. For the other 63 clusters, we randomly picked one protein sequence from each cluster and gathered them as another dataset called Data63. The Data63 is used as an independent test dataset in the study. For both datasets, we randomly selected six non-catalytic residues for one catalytic residue in each sequence. To further compare L1pred and CRpred directly, all chosen methods are compared on the EF-family dataset from [37]. Proteins in this data set that are not the same as or part of the corresponding sequences in PDB were discarded, and 347 chains were left.

### Classifier Feature Vectors

Here, we first describe construction of feature vectors. For a given amino acid residue, we collect a sub-sequence with all residues adjacent to it by a certain window size, e.g. 4, which means the total length of this sub-sequence is 4+1+4 = 9. For this sub-sequence, we encode it with a multidimensional vector based on eight sequence-based attributes. The L1-logreg classifier is then applied to these vectors to train a model and then predict catalytic residues. The eight attributes we use are residue type (RT), overlapping properties (OP), averaged cumulative hydrophobicity (ACH), Jensen-Shannon divergence conservation score (JSD), the combination of relative entropy of Venn diagram and JSD conservation score (VJSD), predicted protein secondary structure (SS), predicted solvent accessible surface area (ASA), and Consurf score. In the following, we describe each attributes in details.

#### Residue Type (RT)

RT is a commonly used attribute for protein-sequence-based machine learning methods. Each amino acid is encoded by a 20-bit binary vector where the dimension of the corresponding amino acids is set to 1 and others are 0, *i.e.*, A (10000000000000000000), … V (00000000000000000001). The order of amino acids in this manuscript is A, R, N, D, C, Q, E, G, H, I, L, K, M, F, P, S, T, W, Y, V.

#### Overlapping Properties (OP)

Several previous studies suggested that the Taylor’s overlapping properties are useful for catalytic residues prediction [8], [19]. These properties are: Polar [NQSDECTKRHYW], Positive [KHR], Negative [DE], Charged [KHRDE], Hydrophobic [AGCTIVLKHFYWM], Aliphatic [IVL], Aromatic [FYWH], Small [PNDTCAGSV], Tiny [ASGC] and Proline [P] [47]. Residues are encoded using 10-bit vectors where the dimensions of the corresponding properties are set to 1 and remaining positions are 0, *i.e.*, A (0000100010), … V (0000110100).

#### Averaged Cumulative Hydrophobicity (ACH)

ACH has been demonstrated to be an important attribute for catalytic residues [37]. The attribute is extracted by computing the average of the cumulative hydrophobicity indices over a window with size varying as 3, 5, 7, …, 21. As a result, ten ACH scores are extracted. Hydrophobicity index proposed by Sweet and Eisenberg [48] is used in the paper. If the central residue is at the sequence termini, we use 0s to fill in the blanks.

#### Jensen-Shannon divergence (JSD) scores

WOP is another important information source extracted by PSI-BLAST [46]. The WOP vector for a position represents the position-specific distribution of 20 amino acids. It has been used to calculate sequence conservation in several previous works [19], [37] and is used as the source of amino acid position-specific distribution in the study. The JSD score of a residue *S* is computed as:
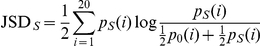


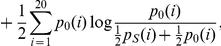
where 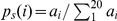, 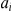 is the 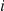
th WOP value at the site (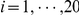) and 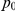 is the BLOSUM62 amino acid background distribution.

#### Combination of relative entropy of Venn diagram and JSD (VJSD)

The relative entropy of Venn diagram (RVD) score is based on Taylor’s Vine diagram of amino acids as shown above in the overlapping properties [19]. Calculating RVD scores needs the WOP matrix from PSI-BLAST as well. The RVD score of the residue on site *S* is defined as:
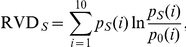
where 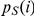 is the fractional WOP values of all residues with the same property 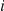 in the site *S*, *i.e.* 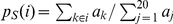, 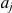 is the 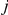
th WOP value, and 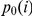 is the fractional BLOSUM62 value of the same class 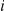 for the background distribution.

Taylor’s Vine diagram can not distinguish residues, such as TYR and TRP, GLY and ALA, ILE and LEU. But methods which based on residue frequencies can discriminate them naturally. Therefore, RVD is combined with JSD, which is based on residue frequencies, to overcome the weakness. The combined score of a residue *S* is given by:
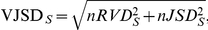
where the 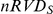 and 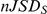 are the normalized RVD and JSD scores of the site.

#### Predicted protein secondary structure (SS)

Previous study suggested that more than 50% catalytic residues occur in coil regions of proteins [43]. Therefore, the protein secondary structure deserves to be considered as an attribute in catalytic residue prediction. The most accurate way to obtain the information of secondary structure would be from the 3D structures of proteins, but for a given protein sequence, currently, we can only predict the secondary structures. In this manuscript, the SS attribute of each residue has three bits to show the possibility scores of three types of secondary structures (H, E, and C), which is predicted by PSIPRED [49].

#### Predicted accessible surface area (ASA)

All catalytic residues are on the surface of enzyme proteins, and hence, large solvent accessibility is also an important feature for the catalytic residues. To improve the prediction accuracy, we combined ASA into our frame as well. For the same reason as for the case of SS, the ASA attribute is also predicted with protein sequences. In this study, we used RVP-net [50] to predict the solvent accessible surface area for each residue for a give protein sequence. Each residue has a value of 0 or 1 for the ASA attribute.

#### Consurf score

The Consurf method is based on evolutionary relations among proteins represented by phylogenetic trees [42]. It was used to predict functional sites of proteins by estimating the degree of sequence conservation among their homologous sequences [51], [52]. Consurf scores of all proteins were obtained from the web server http://consurfdb.tau.ac.il.

When appling the sliding window strategy to a given protein sequence with the above eight scores, we devised a few modifications to circumvent issues. If a residue on a sequence terminus is the central bit of a sliding window, we use 0s to fill in blanks on one side of the window. For attributes RT, OP, and ACH, we just applied them to the central bit of a sliding window, making them independent of the size of the windows.

#### L1-logreg classifier

We use the L1-logreg classifier to score and classify all data vectors, and hence, predict catalytic residues. The classifier is a large-scale solver for L1-regularized logistic regression problems [41], which has been proven to yield models better than those based on unregularized estimations [53]–[55]. For the given data vectors, 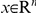, to be classified, the logistic model calculates the conditional probability of 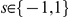,
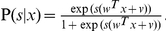



The model has parameters 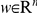 (the weight vector) and 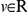 (the intercept); 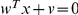 defines the neutral hyper-plane in the data vector space. The classifier locates the optimal model by maximizing the likelihood estimation from the observed examples, *i.e.* minimizing the average logistic loss:
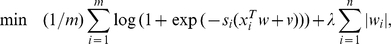
where 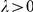 is the regularization parameter, which is used to balance the average logistic loss and the size of the weight vector. More details on the L1-logreg classifier can be found in reference [41]. We used the software package of L1-logreg classifier as implemented by [41] and available at

http://www.stanford.edu/ 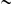
boyd/l1_logreg/.

### Training and Testing Procedure

The parameter 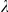 of L1-logreg and the window size were optimized on the dataset Data604 with a ten-fold cross validation. The optimal set of window size and 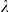 that gives rise to the highest AUPR values, were obtained by a grid search in the interval of [0.001, 0.02] with a step of 0.001 for 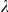 and from 0 to 10 for the window size. For each duplet, the ten-fold cross validation procedure was used to test the performance. Once obtaining the optimal values for window size and 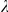, we trained the model on the whole set of Data604 for the test and real prediction. To determine the optimal point of precision and recall rate on the ROC curve, we used the F-measure that is defined in the following equation:
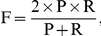
where P and R are Precision and Recall rate, respectively. Please see the section of Evaluation for definitions. We took trained parameters that perform with the maximal F-measure point [56], which is the balance point of sensitivity and specificity.

### Evaluation

To evaluate the performance of our method, we used Precision (P), Recall (R), False Positive Rate (FPR). They are defined by the following equations:
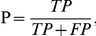


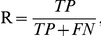


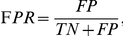
where TP, TN, FP and, FN are the true positive, true negative, false positive, and false negative rate, respectively. To compare among different algorithms, all P, R, and FPR are calculated at the point with the maximal F-measure. The area under the Precision-Recall (PR) curve (AUPR) is also used to evaluate the performances of all methods. A receiver operating characteristic (ROC) curve represents a dependency of sensitivity and (1-specificity). To obtain the ROC curve, all sites in a dataset are sorted by their scores, and we increase the number of predicted sites in steps of one site each time. In addition, the online tool, StAR, is used to test the statistical significance between AUC values [57].
